# Supplementary material for: Mechanical homeostasis of liver sinusoid is involved in the initiation and termination of liver regeneration
Source: Commun Biol. 2021 Apr 7;4:409. doi: 10.1038/s42003-021-01936-2 (PMC8027462; doi:10.1038/s42003-021-01936-2)
Supplement: Supplementary file 2 — Supplementary Information [file 42003_2021_1936_MOESM2_ESM.pdf]

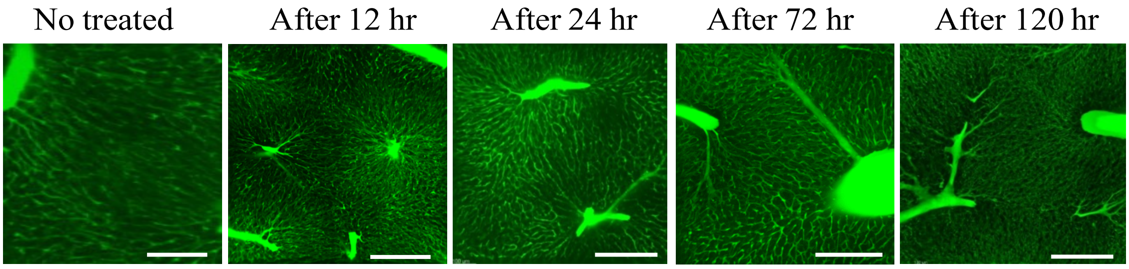

Supplementary Fig. 1 Visualization of sinusoidal structure inside hepatic lobule

Angiographic image obtained using FITC-gelatine in a regenerating liver lobule at indicated time point. Scale bar, 200 μm).

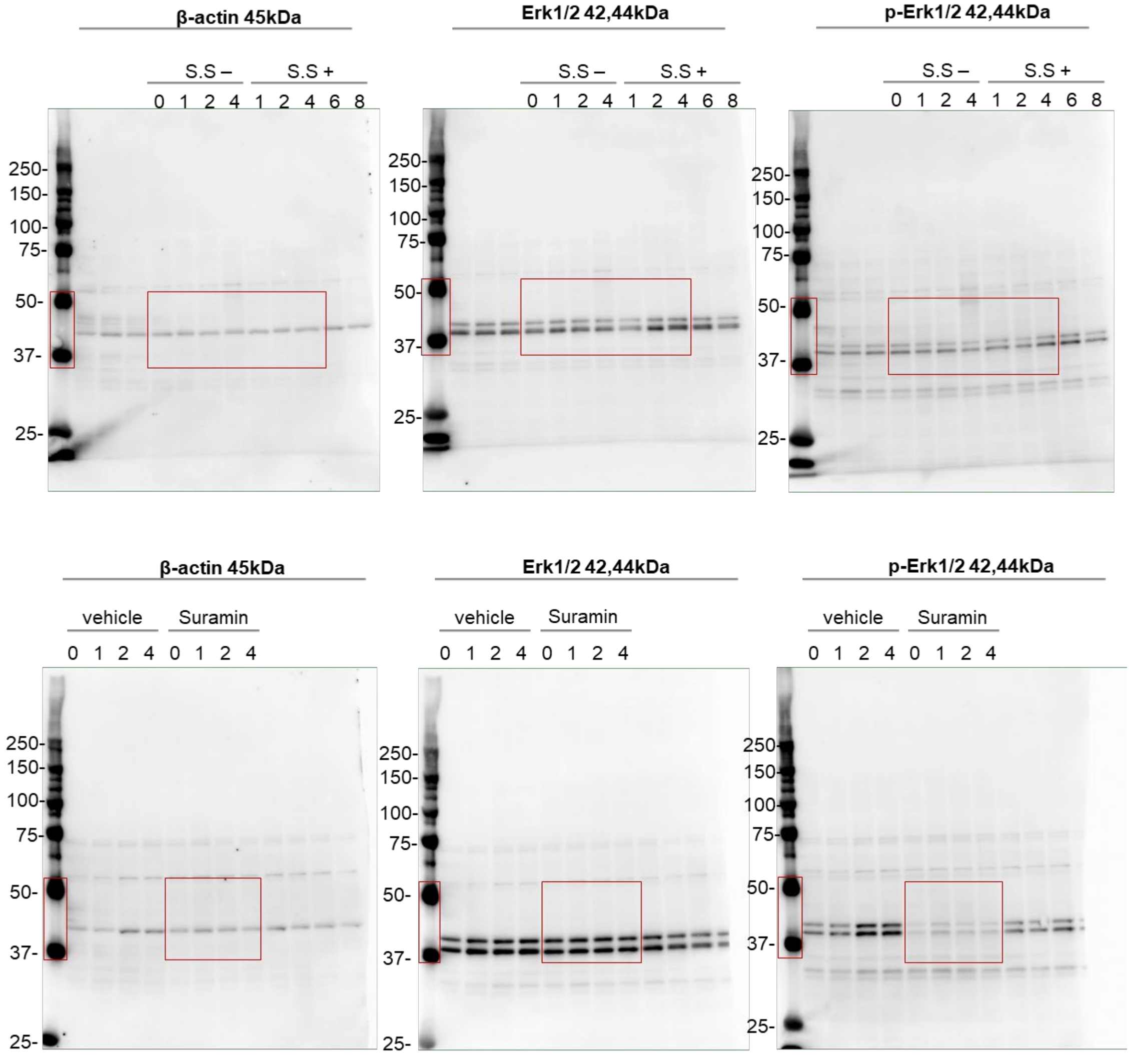

Supplementary Fig. 2 Uncropped western blot image of Erk1/2 phosphorylation using HUVECs

Uncropped western blot image using HUVECs culture with (S.S +) or without shear stress (S.S-) for indicated proteins. Boxed areas are used for main Fig. 4f.

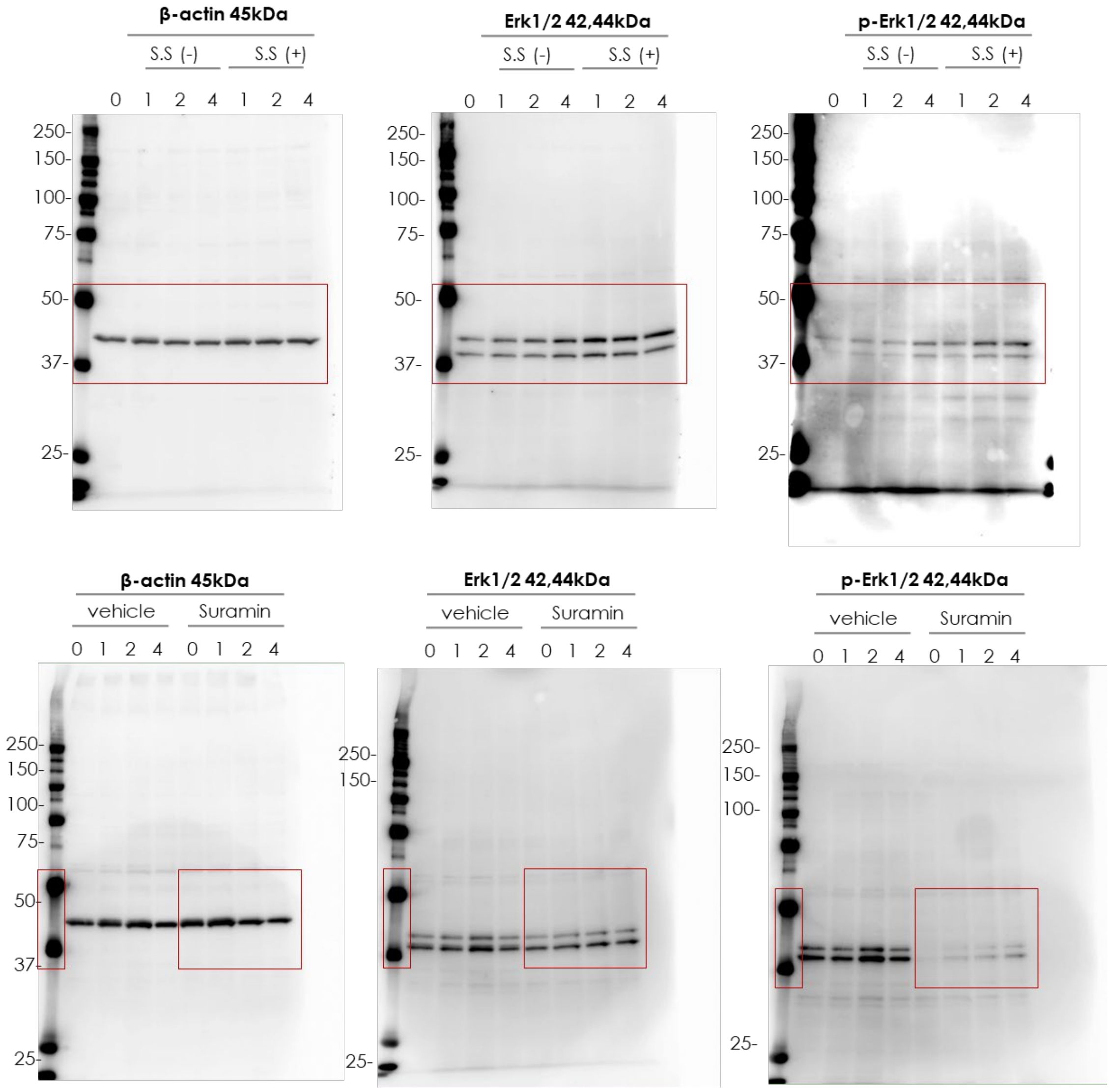

Supplementary Fig. 3 Uncropped western blot image of Erk1/2 phosphorylation using LSECs

Uncropped western blot image using LSECs culture with (S.S +) or without shear stress (S.S-) for indicated proteins. Boxed areas are used for main Fig. 4g.

Supplementary Table 1. Primers for qPCR analysis

	Primer sequence of PCR
rGAPDH 1061 F	TGGCCTCCAAGGAGTAAGAAAC
rGAPDH 1195 R	TGGAATTGTGAGGGAGATGCTC
rCcnd1 665 F	ACATGCACAGACCTTTGTGG
rCcnd1 722 R	TGGGTTGGAAATGAACTTCAC
rTgfb1 468 F	TCACCCGCGTGCTAATGGTG
rTgfb1 606 R	TCTGCACGGGACAGCAATGG
