# Supplementary material for: Mechanical homeostasis of liver sinusoid is involved in the initiation and termination of liver regeneration
Source: Commun Biol. 2021 Apr 7;4:409. doi: 10.1038/s42003-021-01936-2 (PMC8027462; doi:10.1038/s42003-021-01936-2)
Supplement: Supplementary file 3 — Description of Additional Supplementary Files [file 42003_2021_1936_MOESM3_ESM.pdf]

Description of Additional Supplementary Files

File name: Supplementary movie 1

Description: 3D visualization of sinusoidal structure using FITC gelatin

The sinusoid was visualized as green and nuclei were blue. The large blood vessel on the left is the terminal branch of the portal vein and the right is the central vein.

File name: Supplementary movie 2

Description: Vascular linear image of the sinusoidal structure

We obtained the vascular liner image for statistical analysis using Imaris software. We analysed the location, positional relationship, and order and angle of branching of each sinusoid using these images.

File name: Supplementary movie 3

Description: The model of liver vascular structure

We proposed the model of hepatic vascular structure that consisted layered vessels which have different origins.

File name: Supplementary movie 4 - 6

Description: The movement of erythrocytes in the sinusoid

We calculated blood flow velocity from the average moving distance of erythrocytes. The speed of erythrocytes was drastically increased a day after PH (supplementary movie 5) compared to normal (supplementary movie 4), and slightly slowed 3 days after PH (supplementary movie 6).

File name: Supplementary Data 1

Description: The source data for the graphs and charts in the main figures.
