# Supplementary material for: Mechanical homeostasis of liver sinusoid is involved in the initiation and termination of liver regeneration
Source: Commun Biol. 2021 Apr 7;4:409. doi: 10.1038/s42003-021-01936-2 (PMC8027462; doi:10.1038/s42003-021-01936-2)
Supplement: Supplementary file 11 — Reporting Summary [file 42003_2021_1936_MOESM11_ESM.pdf]

nature research

Corresponding author(s): DBPR COMMSBIO-20-2259-T

Last updated by author(s): Feb 8, 2021

Reporting Summary

Nature Research wishes to improve the reproducibility of the work that we publish. This form provides structure for consistency and transparency in reporting. For further information on Nature Research policies, see our Editorial Policies and the Editorial Policy Checklist.

Statistics

For all statistical analyses, confirm that the following items are present in the figure legend, table legend, main text, or Methods section.

n/a	Confirmed	
☐	☒	The exact sample size (*n*) for each experimental group/condition, given as a discrete number and unit of measurement
☐	☒	A statement on whether measurements were taken from distinct samples or whether the same sample was measured repeatedly
☐	☒	The statistical test(s) used AND whether they are one- or two-sided<br>*Only common tests should be described solely by name; describe more complex techniques in the Methods section.*
☐	☒	A description of all covariates tested
☒	☐	A description of any assumptions or corrections, such as tests of normality and adjustment for multiple comparisons
☐	☒	A full description of the statistical parameters including central tendency (e.g. means) or other basic estimates (e.g. regression coefficient) AND variation (e.g. standard deviation) or associated estimates of uncertainty (e.g. confidence intervals)
☐	☒	For null hypothesis testing, the test statistic (e.g. *F*, *t*, *r*) with confidence intervals, effect sizes, degrees of freedom and *P* value noted<br>*Give P values as exact values whenever suitable.*
☒	☐	For Bayesian analysis, information on the choice of priors and Markov chain Monte Carlo settings
☒	☐	For hierarchical and complex designs, identification of the appropriate level for tests and full reporting of outcomes
☒	☐	Estimates of effect sizes (e.g. Cohen's *d*, Pearson's *r*), indicating how they were calculated

Our web collection on statistics for biologists contains articles on many of the points above.

Software and code

Policy information about availability of computer code

Data collection	high-speed recording software (C9100-23B),
Data analysis	Microsoft Excel, Ilmaris 8,

For manuscripts utilizing custom algorithms or software that are central to the research but not yet described in published literature, software must be made available to editors and reviewers. We strongly encourage code deposition in a community repository (e.g. GitHub). See the Nature Research guidelines for submitting code & software for further information.

Data

Policy information about availability of data

All manuscripts must include a data availability statement. This statement should provide the following information, where applicable:

- Accession codes, unique identifiers, or web links for publicly available datasets
- A list of figures that have associated raw data
- A description of any restrictions on data availability

The datasets generated during and analyzed during the current study are available from the corresponding author, Takashi Tsuji, on reasonable request.

Field-specific reporting

Life sciences study design

All studies must disclose on these points even when the disclosure is negative.

Sample size	We stated all sample size in the figure legend.
Data exclusions	We did not exclude any data from the analysis.
Replication	We repeated same experiments at least 3 times and confirm reproducibility.
Randomization	We allocate samples equally and randomly into individual experimental group.
Blinding	We were blinded to sample allocation and during analysis.

Reporting for specific materials, systems and methods

We require information from authors about some types of materials, experimental systems and methods used in many studies. Here, indicate whether each material, system or method listed is relevant to your study. If you are not sure if a list item applies to your research, read the appropriate section before selecting a response.

Materials & experimental systems

n/a	Involved in the study
☐	☒ Antibodies
☒	☐ Eukaryotic cell lines
☒	☐ Palaeontology and archaeology
☐	☒ Animals and other organisms
☒	☐ Human research participants
☒	☐ Clinical data
☒	☐ Dual use research of concern

Methods

n/a	Involved in the study
☒	☐ ChIP-seq
☒	☐ Flow cytometry
☒	☐ MRI-based neuroimaging

Antibodies

Antibodies used	Rat anti-BrdU, ab6326, abcam, Cat#ab6326, lot#GR3173537-5, clone#BU1/75(ICR1)<br>Goat anti-rat IgG antibody, Sigma-Aldrich, Cat#AP183F, Lot# 0507005596<br>Rat anti-CD45 microbeads, Miltenyi biotech, Cat# 130-109-682<br>Mouse anti-PE microbeads, Miltenyi biotech, Cat# 130-105-639<br>anti-CD146 PE-conjugated antibody, Miltenyi biotech, Cat# 130-111-207, Clone#REA697<br>Rabbit anti-p44/42 MAPK antibody, Cell Signaling Technology, Cat# 4695S, Lot#21, Clone#137F5<br>Rabbit anti-phospho-p44/42 MAPK antibody, Cell Signaling Technology, Cat#4370S Lot#,17 Clone#D13.14.4E<br>Mouse anti-beta actin, abcam, Cat#ab8226, Lot#GR231973-4, Clone#mAbcam 8226
Validation	We chose primary antibodies according to the manufacture's validation.

Animals and other organisms

Policy information about studies involving animals; ARRIVE guidelines recommended for reporting animal research

Laboratory animals	We used 5-15w old Male Wistar rats and 6-12w old female C57/BL6 mice
Wild animals	This study did not involve wild animal
Field-collected samples	This study did not involve the samples collected from the field
Ethics oversight	All the experimental procedures using animals were approved by the Institutional Animal Care and Use Committee of RIKEN Kobe Branch

Note that full information on the approval of the study protocol must also be provided in the manuscript.
